# A Comparison of Machine Learning Algorithms for Predicting Hypertension Incidence Based on Cohort Study

**DOI:** 10.1002/edm2.70199

**Published:** 2026-05-10

**Authors:** Somayeh Ghiasi, Susan Darroudi, Mina Moradi, Farzam Kamrani, Muhammad Reza Fatehi, Seyed Masih Sajjadi, Sara Yousefian, Sara Amiri, Majid Ghayour‐Mobarhan, Vahid Mahdavizadeh, Jalal A. Nasiri, Amin Mansoori, Habibollah Esmaily

**Affiliations:** ^1^ Department of Biostatistics, Faculty of Health Mashhad University of Medical Sciences Mashhad Iran; ^2^ Department of Medical and Surgical Sciences for Children and Adults University of Modena and Reggio Emilia Modena Italy; ^3^ Department of Chemistry, Faculty of Science Ferdowsi University of Mashhad Mashhad Iran; ^4^ Department of Nutrition, Faculty of Medicine Mashhad University of Medical Sciences Mashhad Iran; ^5^ Department of Applied Mathematics, Faculty of Mathematical Sciences Ferdowsi University of Mashhad Mashhad Iran; ^6^ Metabolic Syndrome Research Center Mashhad University of Medical Sciences Mashhad Iran; ^7^ Department of Cardiovascular Mashhad University of Medical Sciences Mashhad Iran; ^8^ Social Determinants of Health Research Center Mashhad University of Medical Sciences Mashhad Iran; ^9^ Mathematical Biology Research Laboratory (MBRL), Faculty of Mathematical Sciences Ferdowsi University of Mashhad Mashhad Iran

**Keywords:** hypertension, machine learning, risk factors

## Abstract

**Objectives:**

This study aimed to identify key hypertension (HTN) risk factors using machine learning (ML) models to enhance prediction accuracy.

**Methods:**

Data from the Mashhad stroke and heart atherosclerotic disorder (MASHAD) cohort, comprising 8237 baseline normotensive subjects, was analysed over a 10‐year follow‐up, during which 2548 developed HTN. Five ML algorithms—K‐nearest neighbours (KNN), logistic regression (LR), XGBoost (XGB), random forest (RF) and neural networks (NN)—were employed to determine the best prediction model and identify the primary factors influencing HTN development.

**Results:**

The analysis revealed that the XGBoost model was the most suitable classifier for predicting HTN, outperforming the other algorithms. It achieved the highest AUC‐ROC value (0.79), accuracy (74%), precision of the negative class (86%) and recall of the positive class (74%). Although the precision of the positive class was 55%, and the recall of the negative class was 73%, the XGBoost model demonstrated acceptable performance. Additionally, the ML methods consistently identified age (0.189), copper (0.146), BMI (0.086), triglycerides (0.052), HDL (0.039), glucose (0.039) and uric acid (0.030) as the most influential risk factors, as ranked by SHAP feature importance based on the XGBoost model.

**Conclusion:**

The XGBoost model effectively predicted HTN incidence over 10 years, and age, copper, BMI, triglycerides, HDL, glucose and uric acid were the most significant risk factors. These findings highlight the importance of incorporating ML models into the prediction and prevention of hypertension.

## Graphical abstract

This 10‐year cohort study (MASHAD) including 8237 participants without baseline hypertension used machine learning to predict hypertension incidence. Over follow‐up, 2548 individuals developed hypertension. Among five algorithms tested, XGBoost performed best (AUC‐ROC: 79%, Accuracy: 74%). Key predictors identified by SHAP analysis included copper, BMI, age, triglycerides, uric acid and glucose.

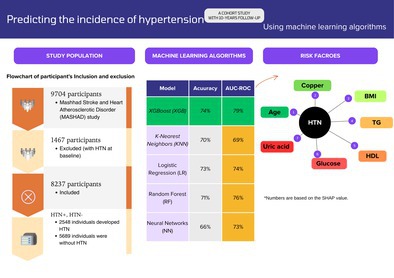

## 1 Introduction

Hypertension (HTN) is a global health issue where a person's blood pressure exceeds 140 mmHg systolic and 90 mmHg diastolic [1]. It is projected that the number of people affected by hypertension worldwide will increase to 1.56 billion by 2025 [2]. According to the World Health Organization (WHO), 31.1% of the global adult population, which is about 1.39 billion people, suffer from hypertension, with two‐thirds of them coming from low and middle‐income countries [3]. The estimated prevalence of hypertension in Iran is 22% [4]. Studies conducted in Iran showed several risk factors contributing to the development of hypertension, including diabetes mellitus, smoking, dyslipidemia, overweight and obesity [5]. Additionally, age [6], depression [7], anxiety [8], uric acid [9] and high sensitive C‐reactive protein (hs‐CRP) [10] are also considered important factors. Some studies have identified Complete Blood Count (CBC) components as predictive indicators for developing hypertension [11]. However, the role of zinc and copper as risk factors for hypertension remains unclear [12]. The prediction of hypertension using these risk factors can be beneficial in identifying individuals at risk and implementing preventive measures.

Machine learning techniques have shown promise in improving disease prediction by analysing large amounts of data and identifying patterns that may not be visible through traditional methods [13]. These techniques have been used in various fields, including finance, retail and especially the healthcare industry [14, 15]. As more data is gathered, the prediction model will learn to make better decisions for accurate predictions [16]. There has been a growing use of machine learning in healthcare, providing more opportunities for disease diagnosis and treatment [17].

Although HTN has several well‐established risk factors, evidence suggests their influence may vary across populations and regions [18]. For instance, in high‐income countries, blood pressure has declined despite increasing body mass index (BMI)—an established risk factor—whereas both BMI and blood pressure continue to rise in most low‐ and middle‐income countries [19], suggesting the importance of further studies in diverse populations. Studies have extensively explored machine‐learning models for hypertension prediction across various regions, highlighting the need for more comprehensive research incorporating diverse factors and methodologies [20]. A recent study in Iran focused solely on anthropometric indicators to predict hypertension, underscoring the importance of considering a broader range of variables [21]. Therefore, conducting hypertension risk prediction research that leverages multiple factors and machine learning methodologies in Iran is crucial. This cohort study, spanning ten years, aims to predict the incidence of hypertension by employing five specific machine learning algorithms: K‐nearest neighbours (KNN), logistic regression (LR), XGBoost (XGB), random forest (RF) and neural networks (NN). The study will also determine the most effective machine learning model for hypertension prediction, providing valuable insights for healthcare professionals and policymakers in Iran.

## 2 Materials and Methods

### 2.1 Study Population

The participants for the study were selected from the Mashhad stroke and heart atherosclerotic disorder (MASHAD) study, which tracked 9704 individuals aged 35 to 65 from 2010 to 2020. They were chosen using a stratified cluster random method from three areas in the North–East of Iran. The MASHAD study excluded individuals with cardiovascular disease (CVD) at the baseline. Since CVD is closely associated with hypertension, excluding patients with CVD at the initial stage would help to more accurately identify the risk factors contributing to the incidence of hypertension. A total of 9704 subjects were followed for 10 years from the baseline. After excluding 1467 participants who had hypertension at baseline, 8237 individuals were included in the analysis [22]. Out of these individuals, 5689 did not develop hypertension after 10 years of follow‐up, while 2548 developed HTN. HTN was defined as SBP ≥ 140 mmHg or DBP ≥ 90 mmHg [23]. The study protocol was approved by the ethics committee of the Mashhad University of Medical Sciences (MUMS) (Code: 85134), and informed consent was obtained from all individuals before their enrollment.

### 2.2 Baseline Examination

Two certified healthcare professionals and a nurse gathered and recorded the demographic, anthropometric and lifestyle data. The stadiometer (SECA 217, Hamburg, Germany) measured the participants' height, waist and hip circumference to the nearest 0.1 cm. Participants' weight was measured using a digital scale, and their BMI was calculated as: dividing weight in kilograms by the square of height in metres.

The participants provided blood and mid‐stream urine samples. Blood samples were obtained between 8 and 10 AM through venepuncture of an antecubital vein after a 14‐h overnight fast. Vacuum tubes (20 mL) were used to collect the blood samples from participants in a sitting position following a standard protocol. All blood specimens were centrifuged at room temperature within 30–45 min of collection to separate the serum and plasma into six aliquots (0.5 mL). The blood concentration of various substances was then measured using standard methods.

Psychometric tests were conducted using Beck's Anxiety Inventory (BAI) to assess anxiety levels [24], and Beck's Depression Inventory II (BDI‐II) was utilised to evaluate depression [25].

### 2.3 Statistical Analysis

The data were analysed using Python 3, Pandas Library (v1.5.3) and stats models Library (v0.14.1). All continuous and normal variables are expressed as mean ± SD (which is determined using the Shapiro–Wilk Test and visual data inspection using QQ‐plot), continuous and abnormal as median (Q1, Q2, Q3), and frequency (%) for categorical variables. The *p*‐value < 0.05 was regarded as statistically significant. A *T*‐test was used to compare continuous and normal variables. The Mann–Whitney U test was applied for continuous abnormal variables, and the chi‐square test was used for categorical variables to compare the mean, median and percent of the subjects of HTN and non‐HTN. The logistic and linear regression were used to compute the odds ratios (OR) and coefficients, respectively, with their 95% confidence interval based on two models. All models include these variables: age, copper, BMI, triglycerides, HDL, glucose, uric acid, uric acid to HDL ratio, LDL, anxiety score, depression score, diabetes, red blood cell (RBC), haematocrit (HCT) test, white blood cell (WBC), hs‐CRP, mean corpuscular volume (MCV), red cell distribution width (RDW), cholesterol, zinc, mean cell haemoglobin (MCH), uric acid to creatinine ratio, platelet distribution width (PDW), neutrophil (Neut), lymphocyte (LYM), Haemoglobin (HGB), creatinine, platelet (PLT), mean platelet volume (MPV), smoking status and sex.

### 2.4 Data Preprocessing

Categorical variables (diabetes, sex, smoking status) were one‐hot encoded, and records with hs‐CRP values exceeding 10 or in the first and last percentiles were removed. The dataset was split into 90/10 training/testing sets. Missing values were imputed using KNN imputer, and continuous features were normalised with standard scaling, both fitted on training data. Full implementation details are available at https://github.com/m‐fatehi/htn_prediction_longitudinal_study.

### 2.5 Machine Learning Models

In our research, we employed a variety of machine learning models to analyse the provided data and predict the target outcome. These models included KNN, LR, XGBoost, RF and NN. Each model offers a unique approach to learning from the data: KNN identifies similar data points to make predictions, LR establishes linear relationships between features and the outcome, XGBoost utilises a powerful ensemble technique for complex predictions, RF leverages multiple decision trees for improved accuracy, and NN mimic the structure of the brain for non‐linear pattern recognition. By employing this diverse set of models, we aimed to comprehensively understand the data and identify the model that delivers the most accurate predictions for the target outcome.

#### 2.5.1 K‐Nearest Neighbours Model

The KNN algorithm is widely recognised as a robust baseline model for binary classification tasks [26]. KNN's simplicity and effectiveness in capturing local patterns within data make it a popular choice for initial experimentation and comparison in research studies. It is a robust benchmark against which more complex algorithms can be evaluated. Moreover, its intuitive nature allows for straightforward interpretation of results, aiding in understanding underlying relationships within medical datasets.

#### 2.5.2 LR Model

LR is a widely utilised statistical method employed for binary classification tasks. Unlike linear regression, LR predicts the probability of occurrence of a binary outcome based on one or more predictor variables. It models the relationship between the independent variables and the probability of the outcome using the logistic function, which ensures that the predicted probabilities fall within the range of 0 and 1. LR provides interpretable coefficients that signify the impact of each predictor variable on the likelihood of the outcome. These coefficients can be exponentiated to obtain OR, representing the change in odds of the outcome for a one‐unit change in the predictor variable. This feature aids in understanding the magnitude and direction of the association between predictor variables and the outcome, enhancing the interpretability of the model within the medical context [27].

#### 2.5.3 RF Model

Ensemble learning is a machine learning technique in which multiple models, often of the same type or different types, are combined to improve predictive performance over any individual model. RF is a powerful ensemble learning technique. It operates by constructing multiple decision trees during the training phase. Each tree in the forest is trained on a random subset of the training data and a random subset of the features, promoting diversity and reducing overfitting. The final prediction is determined by aggregating the predictions of all trees, resulting in a robust and accurate classifier capable of handling large datasets with high‐dimensional feature spaces [28].

#### 2.5.4 XGB Model

Our classification task employed an XGBoost model for extreme gradient boosting. This ensemble learning technique leverages the power of multiple decision trees to create a more robust and accurate predictor. XGBoost builds these trees sequentially, with each new tree focusing on correcting the errors made by the previous ones. This is achieved by employing a gradient boosting framework, where the training process iteratively minimises a loss function that measures the difference between predicted and actual outcomes. XGBoost's strength lies in its ability to handle complex relationships within the data and its effectiveness in reducing overfitting through regularisation techniques. This makes it a powerful tool for various classification problems [29].

#### 2.5.5 NN Model

The second classification algorithm employed in this study was a neural network. NN are inspired by the structure and function of the human brain and consist of interconnected layers of artificial neurons. Each neuron receives weighted inputs from the previous layer, performs a simple mathematical operation on those inputs, and then passes the activation value to the next layer. This allows the network to learn complex, non‐linear relationships between the input features and the desired output classes [30]. In the context of classification, the final layer uses a sigmoid activation function, which transforms the outputs into a probability distribution across the two final classes. This paper used a 4‐layer neural network with 32, 16, 4 and 1 neuron, respectively.

### 2.6 Evaluation

Machine learning models can be evaluated using statistical metrics like area under the receiver operating characteristic curve (AUC‐ROC), precision and recall. These metrics show how well the model classifies data, indicating both successful classifications and mistakes [31].

Due to the limited amount of data available for training, we used 10% of the dataset as a test set. To ensure that both classes were proportionally represented in the training and test sets, we employed stratified sampling based on the target label. This is especially important when dealing with imbalanced datasets, as it ensures that the distribution of classes in the train and test sets reflects the overall distribution, thereby enabling fair evaluation of the model's performance.

To avoid data leakage, all data preprocessing steps—such as imputation and were fitted on the training set only and subsequently applied to the test set. This ensures that information from the test set does not influence the training process. To improve the models' accuracy, we employed GridSearchCV to find the best hyperparameter settings. Additionally, to tune these hyperparameters robustly, we applied k‐fold cross‐validation over the training data.

To assess performance, we consider four key elements:

- True positives (TP): positive cases correctly identified as positive.
- True negatives (TN): negative cases correctly identified as negative.
- False positives (FP): negative cases incorrectly classified as positive (false alarms).
- False negatives (FN): positive cases incorrectly classified as negative (missed cases).

Then, the precision, recall and accuracy metrics are defined as follows:

-
$$\text{Precision}=\frac{\mathrm{TP}}{\mathrm{TP}+\mathrm{FP}}$$
-
$$\text{Recall}=\frac{\mathrm{TP}}{\mathrm{TP}+\mathrm{FN}}$$
-
$$\text{Accuracy}=\frac{\mathrm{TP}+\mathrm{TN}}{\mathrm{TP}+\mathrm{TN}+\mathrm{FP}+\mathrm{FN}}$$
- AUC‐ROC: AUC is the area under the curve. Here, we calculate the area under the ROC curve. The higher the AUC (closer to 1), the better your model is at distinguishing between the two classes. An AUC of 0.5 means your model is no better than random guessing, and 0 means it is completely wrong (always flips positive and negative).

### 2.7 Explanations and Feature Importance

We utilise SHAP values to have integrated explanations between models. SHAP values borrow from game theory to explain how individual features contribute to a model's prediction. Imagine each feature is a player in a team effort, and the prediction is the outcome. SHAP values calculate the average marginal contribution of each feature by considering all possible orders in which features are included in the model. This way, it assigns fair credit to each feature for its impact on the final prediction [32]. In the case of this paper, we use the SHAP python package to calculate the SHAP values of our features, and we can interpret them as the feature importance of a model. We also could use the legacy feature, which comes from some classical machine learning models, but in this way, we cannot have a balanced and fair comparison across different models.

## 3 Results and Model Explanations

### 3.1 Results

#### 3.1.1 Demographic Profile of the Study Cohort

The study involved 8237 individuals, with females comprising 59.6% (4915) and males the remaining 40.4% (3322). Hypertension was diagnosed in 2548 participants (30.9%), while the rest (5689, 69.0%) had normal blood pressure. The mean age across the study was 48 years. However, within the hypertensive group, men were significantly older than women. Table 1 provides further details on these analytical discrepancies. Obesity was a strong indicator of hypertension, with a prevalence rate of 41% compared to 19% in normal‐weight individuals. Similarly, diabetes (51% vs. 27%) was more prevalent among those with hypertension. Statistical analysis revealed significant associations between hypertension and several factors, including age, BMI, glucose, triglycerides, a marker of inflammation (hs‐CRP), cholesterol, lymphocyte count (LYM), red blood cell count (RBC), anxiety score, uric acid, copper levels and red blood cell size variation (RDW). All these associations had a *p*‐value < 0.005.

**TABLE 1:** Baseline characteristics of the study population.

	Total	HTN‐	HTN+	*p*
Count	8237	5689	2548	
Age, years	48.00 (41.00, 54.00)	52.00 (46.00, 58.00)	45.00 (40.00, 52.00)	< 0.001
Sex				
Male	3322 (40.33%)	2334 (41.03%)	988 (38.78%)	0.057
Female	4915 (59.67%)	3355 (58.97%)	1560 (61.22%)	
Smoking				
Non	5622 (68.25%)	3875 (68.11%)	1747 (68.56%)	< 0.001
Former	798 (9.69%)	506 (8.89%)	292 (11.46%)	
Current	1817 (22.06%)	1308 (22.99%)	509 (19.98%)	
BMI, kg/m^2^	27.71 ± 4.27	27.16 ± 4.19	28.95 ± 4.19	< 0.001
Anxiety score	8.00 (3.00, 15.00)	8.00 (3.00, 16.00)	8.00 (3.00, 14.00)	< 0.001
Depression score	10.00 (5.00, 17.00)	11.00 (5.00, 17.00)	10.00 (5.00, 17.00)	0.285
Glucose, mg/dL	82.00 (75.00, 93.00)	86.00 (78.00, 101.00)	81.00 (74.00, 90.00)	< 0.001
Cholesterol, mg/dL	190.83 ± 36.53	188.13 ± 35.78	196.85 ± 37.47	< 0.001
Triglycerides, mg/dL	120.00 (85.00, 171.00)	137.00 (98.00, 191.00)	113.00 (80.00, 161.00)	< 0.001
HDL, mg/dL	41.84 (36.00, 48.30)	41.95 (36.00, 48.00)	41.80 (36.00, 48.50)	0.991
LDL, mg/dL	116.74 ± 34.96	115.55 ± 34.19	119.39 ± 36.48	< 0.001
hs‐CRP, mg/L	1.52 (0.97, 2.88)	1.77 (1.12, 3.37)	1.40 (0.91, 2.62)	< 0.001
Uric acid, mg/dL	4.62 ± 1.25	4.52 ± 1.23	4.85 ± 1.26	< 0.001
BUN, mg/dL	12.75 (11.00, 14.59)	13.00 (11.00, 14.94)	12.62 (11.00, 14.46)	< 0.001
Uric acid to HDL	0.11 (0.08, 0.14)	0.11 (0.09, 0.15)	0.10 (0.08, 0.14)	< 0.001
Uric acid to BUN	0.37 (0.30, 0.44)	0.38 (0.31, 0.45)	0.36 (0.30, 0.44)	< 0.001
Copper, μg/dL	104.00 (80.00, 129.00)	105.00 (79.00, 147.00)	103.00 (80.00, 126.00)	< 0.001
Zinc, μg/dL	78.00 (72.20, 92.00)	78.00 (72.00, 90.92)	78.00 (72.40, 93.30)	0.144
WBC, 10^9^/L	5.80 (5.00, 6.80)	6.00 (5.10, 7.00)	5.80 (5.00, 6.70)	< 0.001
RBC, 10^12^/L	4.85 ± 0.43	4.83 ± 0.43	4.90 ± 0.43	< 0.001
HGB, g/dL	13.80 (12.90, 14.70)	13.80 (13.00, 14.80)	13.70 (12.80, 14.70)	< 0.001
HCT, %	41.25 ± 3.47	41.13 ± 3.51	41.52 ± 3.36	< 0.001
MCV, fL	85.70 (83.10, 88.20)	85.50 (82.90, 88.00)	85.80 (83.20, 88.30)	0.002
MCH, pg/cell	28.80 (27.70, 29.80)	28.70 (27.60, 29.70)	28.85 (27.70, 29.80)	0.004
RDW, %	41.63 ± 2.74	41.69 ± 2.74	41.49 ± 2.72	0.002
PLT, 10^9^/L	228.33 ± 53.76	227.66 ± 53.34	229.82 ± 54.66	0.093
PDW	12.50 (11.40, 13.80)	12.40 (11.40, 13.80)	12.50 (11.40, 13.80)	0.803
MPV, %	10.05 ± 0.88	10.06 ± 0.87	10.03 ± 0.89	0.246
LYM, %	2.10 (1.70, 2.50)	2.10 (1.80, 2.60)	2.00 (1.70, 2.40)	< 0.001
NEUT, %	3.10 (2.50, 3.80)	3.20 (2.60, 3.80)	3.10 (2.50, 3.76)	0.001
Diabetes				
No	7128 (86.54%)	5150 (90.53%)	1978 (77.63%)	< 0.001
Yes	1109 (13.46%)	539 (9.47%)	570 (22.37%)	

#### 3.1.2 Basic Metrics

**FIGURE 1:**
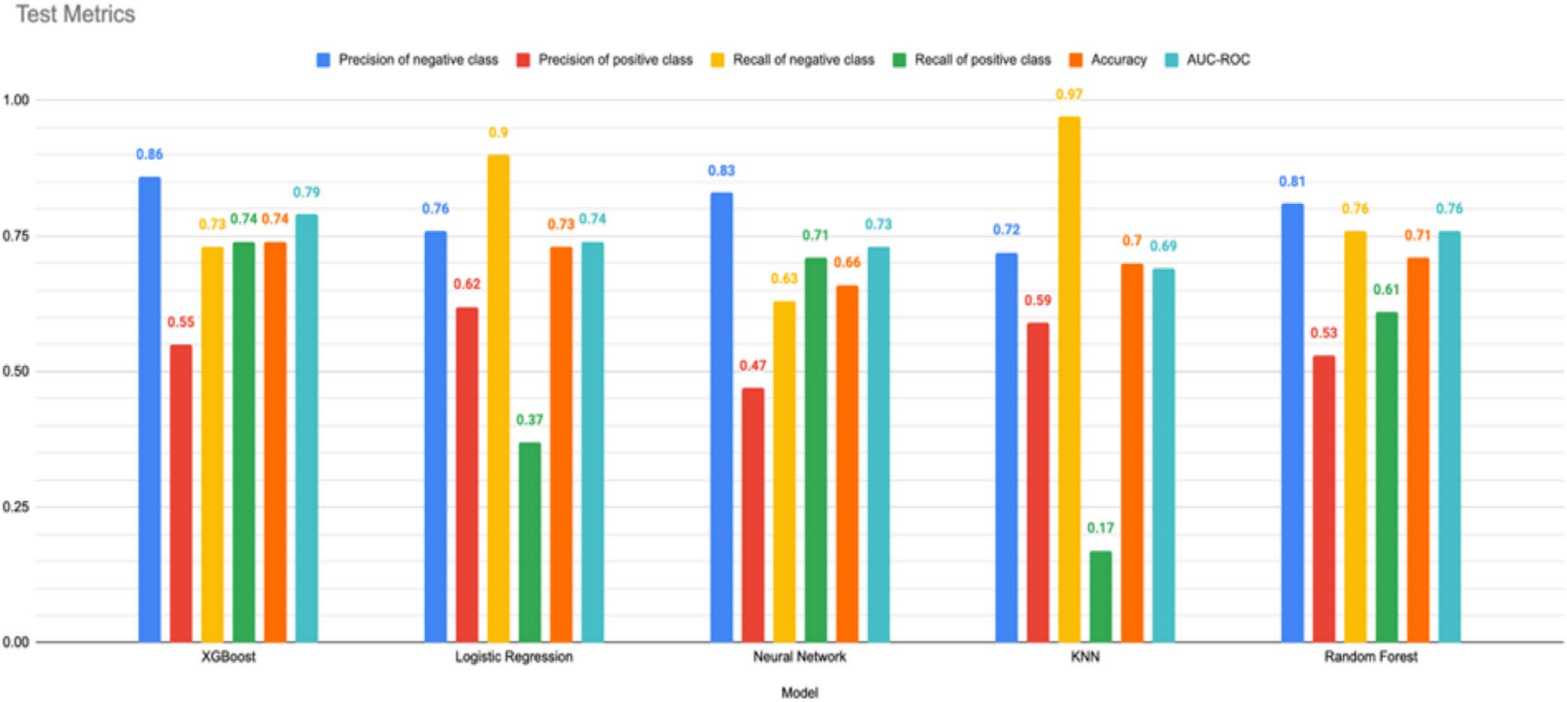
Metrics of train instances: comparison between several machine learning models.

Based on the test metrics (Figure 1), the best model is XGBoost. It achieves the highest overall accuracy (0.74) and demonstrates a good balance between precision and recall for both positive and negative classes. Additionally, its AUC‐ROC score (0.79) is the highest, indicating a strong ability to differentiate between positive and negative instances. While other models might have slight advantages in specific areas, XGBoost's well‐rounded performance across all metrics makes it the optimal choice for this task.

#### 3.1.3 Confusion Matrix

A confusion matrix is like a scorecard for a machine learning model. It helps us understand how well the model is doing beyond just a simple right or wrong answer [33].

**FIGURE 2:**
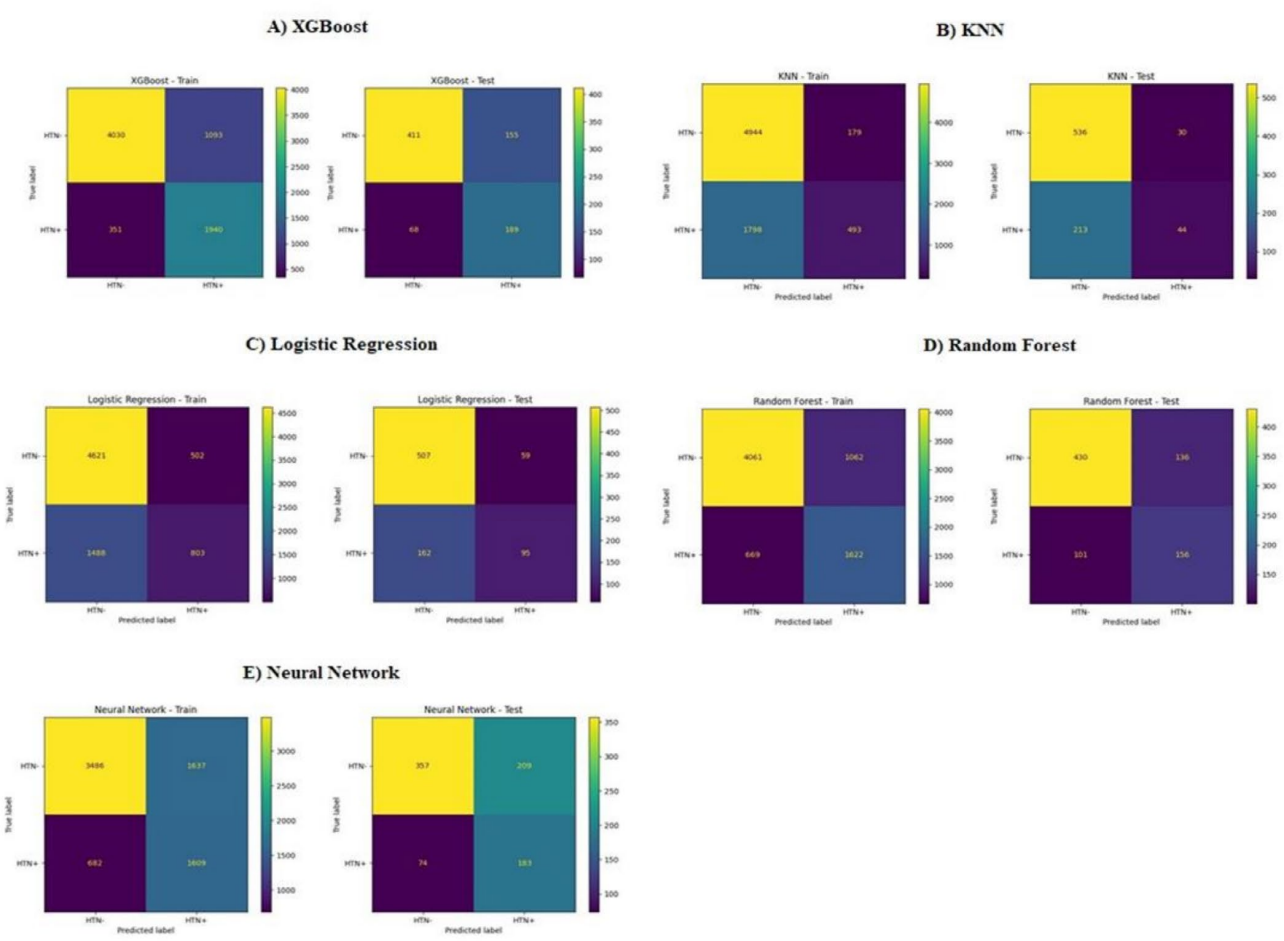
Confusion matrices for models on both train and test data. (A) XGBoost, (B) KNN, (C) logistic regression, (D) random forest, (E) neural network.

Imagine a table with two rows and two columns. Here is what each box tells us (Figure 2):

- Correct guesses:
  ○ Top left: The model correctly predicted something positive, but it actually was positive.
  ○ Bottom right: The model predicted something correctly as negative.
- Incorrect guesses:
  ○ Top right: The model incorrectly predicted something as positive (false positive)—like calling a harmless email spam.
  ○ Bottom left: The model incorrectly predicted something as negative (false negative)—like missing an actual spam email.

As it can be seen in Figure 2, we have the maximum correct (true positive + true negative) predictions in the XGBoost model and minimum wrong (false positive + false negative) predictions in the XGBoost overall. It also confirms the AUC‐ROC score result, which is maximum in the XGBoost model.

### 3.2 Explanations

#### 3.2.1 Normalised SHAP Feature Importance

The normalised feature importance table (Table 2) summarises each feature's contribution to the prediction made by the different machine learning models used in this study. Feature importance scores quantify how much a specific feature influences the model's output. Higher values indicate a greater influence on the model's predictions. In our case, for example, the table shows that ‘age’ has a higher SHAP importance score for the RF model (0.26592) than for the XGBoost model (0.1892). This suggests that while ‘age’ is the most important feature in both models, the RF model places greater reliance on ‘age’ when making predictions than the XGBoost model.

**TABLE 2:** SHAP feature importance ‐ table is sorted based on XGBoost values descending.

Feature	Random forest	KNN	XGBoost	Logistic regression	Neural network
Age	0.265	0.168	0.189	0.25204	0.22674
Copper	0.114	0.037	0.146	0.04471	0.04551
BMI	0.127	0.089	0.086	0.126	0.115
Triglyceride	0.076	0.039	0.052	0.047	0.043
HDL	0.006	0.026	0.039	0.034	0.022
Glucose	0.081	0.040	0.039	0.029	0.032
Uric acid	0.043	0.033	0.030	0.041	0.039
Uric acid to HDL	0.023	0.024	0.027	0.009	0.012
LDL	0.004	0.024	0.025	0.003	0.015
Anxiety score	0.006	0.027	0.022	0.041	0.039
Depression score	0.003	0.024	0.021	0.032	0.017
Diabetes	0.058	0.006	0.020	0.027	0.034
RBC	0.017	0.026	0.020	0.040	0.038
HCT	0.006	0.021	0.020	0.021	0.015
WBC	0.008	0.024	0.019	0.031	0.013
hs‐CRP	0.022	0.029	0.018	0.005	0.008
MCV	0.007	0.021	0.018	0.010	0.008
RDW	0.006	0.030	0.017	0.025	0.026
Cholesterol	0.035	0.028	0.017	0.009	0.014
Zinc	0.004	0.028	0.017	0.014	0.019

#### 3.2.2 SHAP Bee Swarm

SHAP bee swarm contains dots placed in a vertical and horizontal position with a gradient colour ranging from blue to red.

1. Colour speaks for position: The colour of each dot reflects its value within the feature's range, with lower values coded one way and higher values another.
2. Impact on prediction: Dots are spread out horizontally based on how much they influence the outcome. The centre line represents no impact (SHAP value = 0). Dots to the left decrease the outcome, while those to the right increase it.
3. Stacked insights: Dots are stacked vertically. This shows how many data points have a similar level of impact for each feature.

**FIGURE 3:**
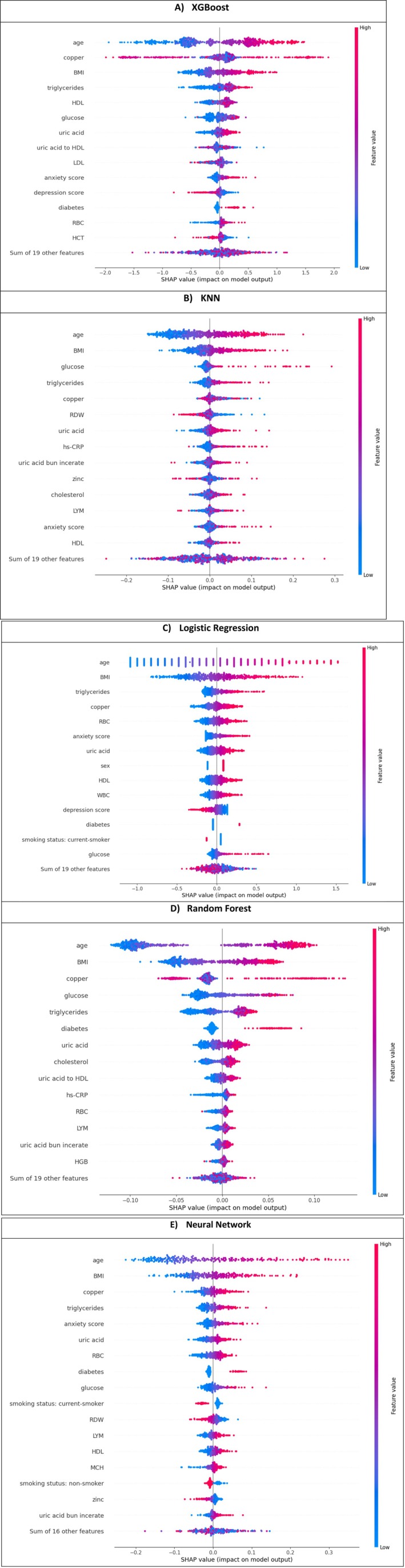
SHAP value of correctly‐predicted test instances on (A) XGBoost, (B) KNN, (C) logistic regression, (D) random forest, (E) neural network.

Our best‐performing model for predicting HTN based on the AUC‐ROC metric, the XGBoost model with SHAP Bee Swarm results, identified age, Copper, BMI, Triglycerides, HDL, Glucose and Uric Acid as the most critical factors influencing HTN risk. This finding aligns with the results in Figure 3 for the neural network model. Looking deeper at the individual factors (Figure 3), we see a strong direct correlation between age and HTN risk. This means that the likelihood of developing HTN increases as age increases. Similarly, high copper levels elevate HTN risk, while low copper levels have no significant impact. Both BMI and Triglycerides also exhibit a direct correlation, with higher values increasing HTN risk and lower values decreasing it. However, the impact of high BMI on HTN risk is more significant than that of high triglycerides. Similar KNN interpretations can be found in Figure 3 (B), RF in Figure 3 (D), LR in Figure 3 (C), XGBoost in Figure 3 (A) and NN in Figure 3 (E).

## 4 Discussion

In the current investigation, various machine‐learning algorithms were studied to create a comprehensive framework for predicting the risk of HTN in Iran. Five algorithms—KNN, LR, XGB, RF and NN—were used and trained to predict HTN using 32 risk factors identified in previous studies. We assessed the models' performance based on accuracy, precision, recall and the ROC curve, with an AUC value on the testing set. After analysing the performance metrics, we found that the XGBoost model was the most suitable classifier for predicting HTN, outperforming the other algorithms. It achieved the highest AUC‐ROC value (0.79), accuracy (74%), precision of the negative class (86%) and recall of the positive class (74%). However, the precision of the positive class was 55%, and the recall of the negative class was 73%. In addition, these machine‐learning methods consistently identified the risk factors with SHAP feature importance based on the XGBoost model, ranking age (0.189), copper (0.146), BMI (0.086), triglycerides (0.052), HDL (0.039), glucose (0.039) and uric acid (0.030) as the most influential factors.

XGBoost has been widely recognised for its effectiveness in predicting CVD and related outcomes [34, 35, 36, 37]. Studies have shown that the XGBoost model consistently outperforms other models regarding performance metrics and precision [36], with one study reporting 98.50% accuracy and 99.14% precision in detecting CVDs [37]. Additionally, the XGBoost model is frequently cited as the best for predicting hypertension, with one study demonstrating an accuracy of 88.81% and a precision of 89.62% [38]. Another study found that XGBoost had the highest accuracy (90%) and recall (100%) for predicting hypertension in three South Asian countries [20].

The comparative findings indicate that age, copper levels, BMI, triglycerides, HDL, glucose and uric acid are significant risk factors associated with the development of HTN. A longitudinal study over 20 years on the prevalence of hypertension among Iranian adults showed a consistently increasing impact of age on hypertension prevalence [39]. This indicates a nearly continuous rise. This trend aligns with the well‐established concept of arterial stiffness, where aging results in stiffening and reduced elasticity in arteries, potentially leading to higher blood pressure. This phenomenon is a significant risk factor for developing hypertension [40]. Further research confirmed this trend by demonstrating that individuals between 70 and 80 years old had a 7.01 times higher prevalence of hypertension compared to those between 40 and 50 years old [41].

Excess body weight can lead to a range of physiological changes that contribute to the development of hypertension. Specifically, it can cause increased insulin resistance, activation of the renin‐angiotensin‐aldosterone system and increased sympathetic nervous system activity, all known risk factors for hypertension [42]. Furthermore, hyperglycemia and dyslipidemia can lead to oxidative stress, inflammation and endothelial dysfunction, which impair vascular function and increase blood pressure [43, 44]. These conditions can also exacerbate hypertension by disrupting the normal functioning of blood vessels and increasing blood pressure. Similarly, studies have consistently identified obesity, diabetes and hyperlipidemia as significant risk factors for hypertension. For example, Katibeh et al. [41] found that obesity was associated with a 2.78‐fold increased risk of hypertension (95% CI: 2.06–3.75), diabetes was associated with a 1.46‐fold increased risk (95% CI: 1.12–1.89), and hyperlipidemia was associated with a 1.60‐fold increased risk (95% CI: 1.26–2.03).

Another factor that can cause oxidative stress is copper. Copper can impact vascular function and contribute to oxidative stress, leading to endothelial dysfunction and increased blood pressure [45, 46]. A study by Darroudi found that individuals with serum copper levels over 130 μg/dL had a 1.94‐fold higher risk of elevated blood pressure. Similarly, participants with serum copper levels below 80 μg/dL had a 1.33 times greater risk of increased blood pressure compared to other participants [47].

Uric acid may lead to endothelial dysfunction, inflammation and activation of the renin‐angiotensin‐aldosterone system, all of which can contribute to increased blood pressure [48]. While many studies have focused on the impact of high levels of uric acid on pulmonary arterial hypertension [49, 50, 51], there is a hypothesis suggesting that elevated serum uric acid is associated with a higher risk of hypertension. Furthermore, a randomised clinical trial conducted by Pour‐Pouneh showed that allopurinol, a drug that lowers uric acid levels, significantly reduced systolic and diastolic blood pressure [52].

### 4.1 Strengths and Limitations

To the best of our knowledge, this is the first study conducted in Iran that determines the factors that predict HTN using machine learning algorithms. We have considered a wide range of risk factors, from CBC components to mental health and demographic characteristics, which can provide valuable insights into the multifactorial way of developing HTN. Furthermore, the 10‐year follow‐up period allows for a deeper investigation of the impact of these risk factors. However, this study has some limitations.

Firstly, the community under examination was exclusively drawn from a distinct cohort in Iran. Furthermore, we regrettably omitted the evaluation of individuals aged below 35 or beyond 65 years, which may conceivably exert an influence on the outcomes.

In addition, while the XGBoost model showed relatively good performance, the overall results reflect moderate classification capability. This may be due to factors such as dataset quality, feature noise or limitations in the available variables that may not fully capture all aspects of HTN risk. The dataset, although valuable, is also limited in size. Since machine learning models typically benefit from larger and more diverse data, future studies with more comprehensive datasets could improve predictive performance. Moreover, the model was trained and evaluated within a single cohort. External validation on independent populations is needed to assess generalizability and clinical applicability. Finally, further research is required to establish causal relationships and confirm these findings across different populations.

## 5 Conclusion

We utilised four distinct machine learning algorithms in our study to create the most suitable predictive model for the classification of HTN. The results of our experiments indicated that, out of the five models, the XGBoost model is the most fitting model for predicting patients with the risk of HTN. After conducting a SHAP analysis, we found that age, copper, BMI, triglycerides, HDL, glucose and uric acid are the risk factors contributing to the development of HTN. The proposed integrated system can be easily used as a valuable tool in clinical settings to accurately identify patients with the risk of HTN at an early stage. With this information, physicians can make decisions that will reduce healthcare costs and time while also enabling them to provide individualised interventions and targeted treatment to minimise the burden of HTN in Iran.

## Author Contributions

Somayeh Ghiasi, Amin Mansoori, Seyed Masih Sajjadi, Mohammad Reza Fatehi: formal analysis, software. Susan Darroudi, Somayeh Ghasi, Farzam Kamrani: writing – original draft. Amin Mansoori, Vahid Mahdavizadeh, Mina Moradi: writing – review and editing. Sara yousefian, Sara Amiri, Jalal A. Nasiri: methodology. Habibollah Esmaily, Amin Mansoori: supervision. Majid Ghayour‐Mobarhan: conceptualization. All authors read and approved the final manuscript.

## Ethics Statement

Accordingly, the study protocol was validated by the Ethics Committee of the Mashhad University of Medical Sciences (MUMS) and the Institutional Review Board of Mashhad University Medical Center. Mashhad University of Medical Sciences supports this project. Funding number: 85134 and ethical approval code: IR.MUMS.REC.1386.250.

## Consent

Informed consent was obtained from all subjects.

## Conflicts of Interest

The authors declare no conflicts of interest.

## Data Availability

Data cannot be shared due to privacy. However, the datasets used and/or analysed during the current study are available from the corresponding author upon reasonable request.
